# Resurrection of sildenafil: potential for Huntington’s Disease, too?

**DOI:** 10.1007/s00415-022-11196-7

**Published:** 2022-05-28

**Authors:** Jannis Achenbach, Simon Faissner, Carsten Saft

**Affiliations:** Department of Neurology, Huntington Center North Rhine-Westphalia, Ruhr-University Bochum, St. Josef-Hospital Bochum, Gudrunstraße 56, 44791 Bochum, Germany

**Keywords:** Huntington’s disease, Neurodegeneration, Sildenafil, Phosphodiesterase-inhibitor

## Abstract

The phosphodiesterase-5 inhibitor sildenafil was postulated to reduce the risk for Alzheimer’s Disease. Since preclinical data revealed beneficial effects in Huntington’s Disease (HD), we now for the first time investigated effects of sildenafil in HD patients using the database ENROLL-HD. We demonstrate beneficial effects on motoric, functional and cognitive capacities in cross-sectional data. Those effects were not explained by underlying fundamental molecular genetic or demographic data. It remains unsolved, if effects are due to behavioral differences or due to direct dose-dependent neurobiological modulations.

## Introduction

Recently, Fang et al. performed a pharmacoepidemiologic analysis of data from 7.23 million individuals published in *Nature Aging* and found, that treatment with sildenafil leads to a 69% risk reduction for developing Alzheimer’s Disease (AD). In addition the authors performed mechanistic analyses and found that sildenafil leads to an increased neurite growth and decreased phospho-tau expression in neuron models [1].

Inspired by this research, we set out to investigate potential effects of sildenafil in another neurodegenerative disease, Huntington’s Disease (HD). HD is a neurodegenerative, autosomal-dominant inherited disorder, accompanied by progressive motoric, cognitive and behavioral-psychiatric decline [2, 3]. Until now, no disease modifying or causal therapy is available, stressing the urgent need for identifying and developing new targets and therapeutics [4, 5]. The vasodilating phosphodiesterase-5 (PDE-5) inhibitor sildenafil, licensed for erectile dysfunction (ED), positively modulates neurodegenerative processes in AD through regulation of cyclic guanosine monophosphate (cGMP) and cyclic adenosine monophosphate (cAMP) in signaling pathways of neurons [6–8]. Further potential pathways modulated by sildenafil in AD include vascular endothelial growth factor (VEGF) and vascular cell adhesion molecule-1 (VCAM-1) as well as α-synuclein accumulation [9].

In HD, the regulation of hippocampal cGMP levels with sildenafil was shown earlier to beneficially modulate cognitive decline in a preclinical HD model [10]. More specifically, the negative role of mutant huntingtin protein on transcription factors, leading to cAMP response element-binding protein (CREB) and brain-derived neurotrophic factor (BDNF) changes, might be positively targeted by PDE inhibitors [11, 12]. Two animal studies described beneficial biochemical and behavioral effects of sildenafil in the 3-nitropropionic acid (3-NP) induced experimental model of HD [12, 13].

In summary, those mechanistic data derived from AD and preclinical HD models support the hypothesis that sildenafil might elicit positive effects in HD regarding both onset of the disease and clinical progression. The aim of this study was to investigate effects of treatment with sildenafil in HD patients regarding clinical characteristics and disease modifying effects in a large real-world cohort of HD patients.

The world-wide registry study ENROLL-HD provides the largest prospectively followed HD cohort. We analyzed data of *n* = 21,116 participants, whereby *n* = 47 males met inclusion criteria for manifest HD treated with sildenafil compared to *n* = 5261 other manifest male patients. Indications for treatment initiation were as follows: *n* = 41 erectile dysfunction, *n* = 3 hypertension, *n* = 2 sexual dysfunction and *n* = 1 libido decrease. Frequency of intake revealed that *n* = 37 reported about intake as needed, *n* = 6 about daily intake, *n* = 3 about weekly intake and *n* = 1 about every 2nd day intake, all p.o. Values of total daily intake dose revealed in mean 81.1 mg (SD 64.9; range 20–400). The start date of intake transformed into days prior to baseline revealed a medium intake of 1354 days (SD 1478; range 5796–13). Between groups, no differences were observed regarding sociodemographic parameters of age, genetics and onset of HD except for educational level which was afterwards implemented as a co-variate to avoid a potential bias (Table 1). To test robustness of obtained results, we additionally performed a 1:3 propensity-score matching controlling for variables age, gender, education, CAG-high, disease duration, onset of HD motor symptoms, PBA-depression score and matched manifest control patients to those with sildenafil intake prior to baseline visit having two more follow-up visits.

**Table 1:** Demographic and clinical characteristics between groups

	Manifest HD treated with sildenafil (*n* = 47)	Manifest HD control group (*n* = 5261)	*F*	*P*	Part. Eta^2^
Age (y); M (SD)	54.6 (12.4)	52.9 (12.8)	0.803	0.370	0.000
CAG high	43.1 (2.9)	43.9 (3.9)	2.554	0.110	0.000
Motoric onset	48.8 (12.4)	45.9 (12.6)	2.304	0.129	0.000
ISCED	4.2 (0.9)	3.4 (1.2) (*n* = 5240)	18.430	< 0.001	0.003
UHDRS TMS; M (SD) #	30.1 (16.3)	36.3 (20.9)	3.985	< 0.050	0.001
TFC +	9.9 (2.7)	8.5 (3.6)	4.053	< 0.050	0.001
IS +	84.8 (14.2)	78.5 (18.4) (*n* = 5227)	2.422	0.120	0.000
SDMT +	29.4 (11.3) (*n* = 46)	23.3 (12.7) (*n* = 4827)	4.813	< 0.050	0.001
Verfct +	14.2 (5.8) (*n* = 46)	12.2 (5.8) (*n* = 5056)	2.257	0.133	0.000
SCNT +	53.1 (16.0) (*n* = 46)	42.3 (17.9) (*n* = 4961)	10.288	< 0.005	0.002
SWRT +	68.1 (21.8) (*n* = 46)	56.4 (23.4) (*n* = 4959)	5.940	< 0.050	0.001
SIT +	27.5 (11.2) (*n* = 42)	24.1 (11.6) (*n* = 4285)	1.199	0.274	0.000
VerFc +	28.8 (14.2) (*n* = 31)	21.2 (12.8) (*n* = 3765)	5.180	< 0.050	0.001
MMSE +	26.9 (2.4) (*n* = 23)	25.1 (4.3) (*n* = 3285)	1.877	0.171	0.001

+ : Higher scores = better performance; #: Higher scores = more impairment

*CAG* Cytosine-Adenine-Guanine repeat length, *ISCED* Educational level, *UHDRS* Unified Huntington’s Disease Rating Scale, *TMS* Total motor score, *TFC* Total functional capacity, *IS* Independence scale, *SDMT* Symbol digit modality test, *Verfct* Verbal fluency test (category), *SCNT* Stroop color naming test, *SWRT* Stroop word reading test, *SIT* Stroop interference test, *VerFc* Verbal Fluency Test (Letters), *MMSE* Mini mental state examination

The cross-sectional analysis revealed that HD patients treated with sildenafil had less motoric and functional impairments as well as better cognitive capacities in four tests (Symbol digit modality test; Stroop color naming test; Stroop word reading test; Verbal fluency test) compared to other motor-manifest HD (all < 0.050; Table 1). The conducted additional case–control approach confirmed that patients medicated with sildenafil revealed better cognitive capacities in five out of seven cognitive tests in the cross-sectional analysis (Table 2).

**Table 2:** Confirmatory 1:3-Propensity score analysis using variables age, gender, education, CAG-high, disease duration, onset of HD motor symptoms, PBA-depression score

	Manifest HD treated with sildenafil (*n* = 25)	Manifest HD control group (*n* = 75)	*F*	*P*	Part. Eta^2^
Age (y); M (SD)	57.3 (10.8)	61.2 (11.0)	2.458	0.120	0.024
CAG high	43.0 (2.6)	42.3 (2.2)	1.567	0.214	0.016
Motoric onset	50.3 (10.2)	53.6 (10.1)	1.908	0.170	0.019
Disease duration	6.9 (4.6)	7.7 (4.6)	0.485	0.488	0.005
PBA Depression	5.4 (7.0)	3.6 (5.4)	1.770	0.186	0.018
ISCED	4.3 (0.9)	4.1 (1.2)	0.150	0.700	0.002
UHDRS TMS; M (SD) #	34.3 (17.0)	39.6 (17.1)	1.767	0.187	0.018
TFC +	9.4 (3.2)	8.0 (3.4) (*n* = 73)	2.820	0.096	0.029
IS +	82.4 (16.6)	76.3 (16.7)	2.526	0.115	0.025
SDMT +	27.4 (10.6) (*n* = 24)	20.5 (11.3) (*n* = 71)	6.944	< 0.050	0.069
Verfct +	13.9 (4.4) (*n* = 24)	10.6 (5.1) (*n* = 73)	7.982	< 0.050	0.078
SCNT +	49.8 (13.8) (*n* = 24)	35.7 (14.1) (*n* = 74)	18.403	< 0.001	0.161
SWRT +	65.3 (19.8) (*n* = 24)	48.9 (20.9) (*n* = 74)	11.472	< 0.005	0.107
SIT +	26.5 (11.5) (*n* = 22)	20.3 (10.6) (*n* = 60)	5.151	< 0.050	0.060
VerFc +	24.0 (10.9) (*n* = 17)	20.3 (11.7) (*n* = 50)	1.312	0.256	0.020
MMSE +	27.1 (2.8) (*n* = 12)	25.0 (5.1) (*n* = 38)	1.78	0.186	0.036

+ : Higher scores = better performance; #: Higher scores = more impairment

*CAG* Cytosine-Adenine-Guanine repeat length, *PBA* Problem Behaviours Assessment-Short Depression Scale, *ISCED* Educational level, *UHDRS* Unified Huntington’s Disease Rating Scale, *TMS* total motor score, *TFC* total functional capacity, *IS* Independence scale, *SDMT* Symbol digit modality test, *Verfct* Verbal fluency test (category), *SCNT* Stroop color naming test, *SWRT* Stroop word reading test, *SIT* Stroop interference test, *VerFc* Verbal Fluency Test (Letters), *MMSE* Mini mental state examination

Longitudinally and with regard to functional parameters we identified that sildenafil intaking patients remarkably had a significantly lower decrease of impairment in the Independence scale (*p* < 0.050) over time. Cognitive capacities differed significantly over time between both groups in five out of seven tests. However, cognitive data were inconsistent, with no distinct beneficial or worsening effect in the sildenafil group compared to controls. Patients from the sildenafil group had a beneficial effect with less worsening in the Symbol digit modality and Verbal fluency test, though manifest control group had less worsening in the SCNT, SWRT and SIT if compared to the appropriate other group (Table 3).

**Table 3:** Longitudinal analyses of motoric, functional and cognitive parameters

	Manifest HD treated with sildenafil (*n* = 25)				Manifest HD control group (*n* = 75)				*F*	*P*	Part. Eta^2^
	BL	FU 1	FU 2	∆FU2-BL Per group	BL	FU1	FU2	∆FU2-BL per group			
UHDRS TMS; M (SD) #	33.7 (17.1) (*n* = 24)	38.5 (19.9)	41.8 (22.8)	*8.1*	40.8 (16.8) (*n* = 68)	43.6 (20.9)	48.3 (21.1)	*7.5*	1.890	0.173	0.021
TFC +	9.0 (3.1) (*n* = 23)	8.0 (3.1)	8.3 (3.3)	*− 0.7*	7.8 (3.4) (*n* = 67)	7.1 (3.6)	6.0 (3.4)	*− 1.8*	3.551	0.063	0.039
IS +	81.7 (16.6) (*n* = 24)	78.5 (15.9)	77.1 (16.0)	− 4.6 *	75.2 (15.8) (*n* = 69)	72.2 (16.8)	66.9 (17.2)	*− 8.3*	4.137	< 0.050	0.043
SDMT +	27.1 (10.7) (*n* = 23)	26.8 (11.6)	26.9 (12.4)	− 0.2 *	22.3 (10.0) (*n* = 53)	20.7 (9.2)	18.3 (10.1)	*− 4.0*	6.785	< 0.050	0.084
Verfct +	13.9 (4.5) (*n* = 23)	13.5 (5.3)	13.5 (5.6)	− 0.4 **	10.8 (4.4) (*n* = 61)	9.7 (4.4)	9.9 (5.2)	*− 0.9*	10.669	< 0.005	0.115
SCNT +	49.7 (14.1) (*n* = 23)	46.7 (16.0)	43.5 (17.4)	*− 6.2*	37.7 (13.1) (*n* = 60)	36.4 (16.7)	33.1 (13.0)	− 4.6 **	10.856	< 0.005	0.118
SWRT +	64.9 (20.1) (*n* = 23)	60.8 (19.1)	56.2 (24.2)	*− 8.7*	51.6 (18.1) (*n* = 59)	47.0 (18.0)	45.8 (17.7)	− 5.8 *	7.857	< 0.050	0.089
SIT +	27.1 (11.7) (*n* = 20)	26.9 (11.2)	25.0 (12.9)	*− 2.1*	21.3 (8.6) (*n* = 46)	19.6 (8.8)	19.5 (9.2)	− 1.8 *	6.011	< 0.050	0.086
Verflt +	24.9 (11.1) (*n* = 15)	25.8 (12.4)	26.2 (12.7)	*1.3*	20.6 (10.8) (*n* = 35)	20.3 (10.9)	18.6 (10.7)	*− 2.0*	3.184	0.081	0.062
MMSE +	27.3 (2.9) (*n* = 9)	26.8 (3.9)	27.7 (3.0)	*0.4*	24.9 (4.8) (*n* = 26)	24.6 (4.5)	23.7 (6.1)	*− 1.2*	2.583	0.118	0.073

Data were analyzed using repeated measures analysis of variance between groups at baseline and two more follow up visits. Data depicted as mean performance levels (standard deviation) in groups. + : Higher scores = better performance; #: Higher scores = more impairment. *Significant beneficial (*p < 0.050; **p < 0.005) inter-subject effects between groups based on the marked group

*UHDRS* Unified Huntington’s Disease Rating Scale, *TMS* total motor score, *TFC* total functional capacity, *IS* Independence scale, *SDMT* symbol digit modality test, *Verfct* verbal fluency test (category), *SCNT* Stroop color naming test, *SWRT* Stroop word reading test, *SIT* Stroop interference test, *VerFc* Verbal Fluency Test (Letters), *MMSE* Mini mental state examination

As evaluated in cross-sectional data in the comparison with matched control patients, sildenafil intaking patients revealed better cognitive capacities during baseline visit. Thus, as a potential explanation for divergent findings in longitudinal results of cognitive tests, different baseline capacities might have had an influencing effect. To control for these intergroup effects, we additionally regarded intragroup effects, revealing that both groups significantly decreased in motoric capacities over time between baseline and second follow-up visit. Regarding cognitive tests, manifest HD patients treated with sildenafil showed a decrease over time in only two out of seven cognitive tests (SCNT, SWRT) whereas the matched control group significantly decreased in four out of seven tests (Table 4). Thus, not only cross-sectional, but also some longitudinal data support the hypothesis of a beneficial effect on motoric, functional and cognitive capacities in patients medicated with sildenafil. Those findings, however, need further evaluation of comparable beneficial longitudinal effects. Longitudinal data in this context are more difficult to interpret because of different baseline findings between groups, potentially leading to well-known ceiling effects for motor and cognitive scales and thus reduced sensitivity for worsening over time in the more affected control group [14]. Thus, our finding of less functional worsening over time might be more relevant in this longitudinal analysis.

**Table 4:** Longitudinal analyses of inner-subject parameters

	Manifest HD treated with sildenafil (*n* = 25)			*F*	*P*	Part. Eta^2^
	BL	FU 2	∆FU2-BL per group			
UHDRS TMS; M (SD) #	33.7 (17.1) (*n* = 24)	41.8 (22.8)	*8.1*	17.719	< 0.001	0.425
TFC +	9.0 (3.1) (*n* = 23)	8.3 (3.3)	*− 0.7*	3.794	0.063	0.137
IS +	81.7 (16.6) (*n* = 24)	77.1 (16.0)	*− 4.6*	6.205	0.020	0.205
SDMT +	27.1 (10.7) (*n* = 23)	26.9 (12.4)	*− 0.2*	0.066	0.799	0.003
Verfct +	13.9 (4.5) (*n* = 23)	13.5 (5.6)	*− 0.4*	0.202	0.657	0.009
SCNT +	49.7 (14.1) (*n* = 23)	43.5 (17.4)	*− 6.2*	12.061	< 0.005	0.344
SWRT +	64.9 (20.1) (*n* = 23)	56.2 (24.2)	*− 8.7*	10.621	< 0.005	0.316
SIT +	27.1 (11.7) (*n* = 20)	25.0 (12.9)	*− 2.1*	4.098	0.056	0.170
Verflt +	24.9 (11.1) (*n* = 15)	26.2 (12.7)	*1.3*	0.359	0.558	0.023
MMSE +	27.3 (2.9) (*n* = 9)	27.7 (3.0)	*0.4*	0.028	0.871	0.003

	Manifest HD control group (*n* = 75)			*F*	*P*	*Part. Eta*.^*2*^
	BL	FU2	*∆* *FU2-BL* *per group*			
UHDRS TMS; M (SD) #	40.8 (16.8) (*n* = 68)	48.3 (21.1)	*7.5*	37.344	< 0.001	0.338
TFC +	7.8 (3.4) (*n* = 67)	6.0 (3.4)	*− 1.8*	49.662	< 0.001	0.408
IS +	75.2 (15.8) (*n* = 69)	66.9 (17.2)	*− 8.3*	52.769	< 0.001	0.416
SDMT +	22.3 (10.0) (*n* = 53)	18.3 (10.1)	*− 4.0*	34.933	< 0.001	0.368
Verfct +	10.8 (4.4) (*n* = 61)	9.9 (5.2)	*− 0.9*	2.497	0.119	0.036
SCNT +	37.7 (13.1) (*n* = 60)	33.1 (13.0)	*− 4.6*	4.676	< 0.050	0.065
SWRT +	51.6 (18.1) (*n* = 59)	45.8 (17.7)	*− 5.8*	8.083	< 0.050	0.109
SIT +	21.3 (8.6) (*n* = 46)	19.5 (9.2)	*− 1.8*	3.541	0.066	0.065
Verflt +	20.6 (10.8) (*n* = 35)	18.6 (10.7)	*− 2.0*	3.376	0.074	0.078
MMSE +	24.9 (4.8) (*n* = 26)	23.7 (6.1)	*− 1.2*	4.380	< 0.050	0.135

Data were analyzed using repeated measures analysis of variance between baseline and the second follow up visit in the sildenafil and control group. Data depicted as mean performance levels (standard deviation) in groups. + : Higher scores = better performance; #: Higher scores = more impairment

*UHDRS* Unified Huntington's Disease Rating Scale, *TMS* total motor score, *TFC* total functional capacity, *IS* independence scale, *SDMT* symbol digit modality test, *Verfct* verbal fluency test (category), *SCNT* Stroop color naming test, *SWRT* Stroop word reading test, *SIT* Stroop interference test, *VerFc* Verbal Fluency Test (Letters), *MMSE* Mini mental state examination

To the best of our knowledge, this is the first investigation having analyzed sildenafil intake in clinical HD. Inspired by evidence from AD postulating sildenafil, commonly and for many years in clinical use to treat ED, has potential effects against progression and leads to risk-reduction, we set out to analyze effects in HD [1, 8, 15]. Remarkably, data revealed less motoric (TMS) and functional (TFC) impairments of sildenafil treated patients and better cognitive performances. These findings are in line with preclinical findings, postulating sildenafil to increase hippocampal cGMP levels as a potential strategy against cognitive decline [10]. One potential explanation for a detection of a potential therapeutic effect of sildenafil in the patient cohort analyzed here might be that patients had long lasting and regular intake, which might have led to continuously higher cGMP levels with positive effects on cognition.

As a limitation, this hypothesis, however, needs further validation, since other unknown effects within the investigated groups might have had an undetected influence and number of patients on sildenafil was still low even if data are from the worldwide biggest database for HD available today. Certainly, it cannot be determined whether effects can be interpreted as a dose–response or whether or not the group of possibly more or still sexually active sildenafil-intaking patients might have had more functional, motoric, and cognitive abilities as a biasing factor. This, however, remains unclear since no surveys about the sexual-activity and dysfunction in HD are included within the dataset [16]. Questionnaires like these might help to get a better systematical understanding of other influences on HD and, e.g., verify a hypothesis coming from Parkinson’s Disease (PD) research, postulating intact sexual activity in affected patients might correlate with better non-motor and motoric outcomes [17]. This hypothesis might be an alternative explanation for our data, documenting sildenafil-intaking patients having less motoric and functional impairments and better cognitive capacities. Indeed, sildenafil treated patients had a slightly later motoric onset of 2.9 years, which was not significant but showed a trend; potentially explaining the effect. Although controlling for education as a potential influencing factor, we cannot exclude, that the higher cognitive capacities in our sildenafil group are at least partly caused by slightly higher educational levels. Higher educational levels, however, would not explain having less motoric impairments. To assess dose-dependent effects of sildenafil in HD in more detail, prospective double-blinded interventional studies are necessary.

In summary, we show potential beneficial effects of sildenafil intake on disease manifestation of HD via analysis of cross-sectional and longitudinal real-world data of the world-wide largest HD cohort. Those effects were not explained by underlying fundamental molecular genetic or demographic data. It remains unsolved, whether differences might be related to molecular effects of sildenafil or whether patients might have been less impaired by HD.

## Methods

We investigated the worldwide registry study ENROLL-HD to identify manifest HD patients treated with sildenafil and compared onset, motoric, functional and cognitive cross-sectional data to motor-manifest HD patients without sildenafil treatment. Enroll-HD is a global clinical research platform designed to facilitate clinical research in HD. Core datasets are collected annually from all research participants as part of this global multi-center longitudinal observational study. Data are monitored for quality and accuracy using a risk-based monitoring approach. All sites are required to obtain and maintain local ethical approval. We investigated the periodic dataset five (PDS5) as previously described [18, 19]. Ethics approval was obtained by the local ethics committee of Ruhr-University Bochum (No. 4941-14).

As inclusion criteria for manifest HD group, all participants had a diagnostic confidence level (DCL) of 4 (unequivocal signs of clinical manifest HD (> 99% confidence), a total motor-score (TMS) > 5 and a genetically confirmed report with ≥ 36 Cytosine-Adenine-Guanine (CAG)-repeats in the Huntingtin-gene (*HTT*). Groups were formed due to sildenafil intake. Fundamental demographic and molecular-genetic parameters were assessed analyzing CAG-repeat lengths, age, educational level, age at HD diagnosis, age at onset of symptoms reported by the patient, family and rater between groups. Motoric parameters were analyzed using the UHDRS—Total motor score (TMS). Cognitive performance was evaluated with the ENROLL-HD test battery including seven cognitive tests: Symbol digit modality test (SDMT), Verbal fluency test (category; Verfct), Verbal Fluency Test (Letters; VerFc), Stroop color naming (SCN), Stroop-word reading (SWR), Stroop interference test (SIT) and Mini mental state examination (MMSE). Functionality was analyzed with the UHDRS-Total functional capacity (TFC) and Independence Scale (IS).

Group means and standard deviation for cross-sectional data were assessed using univariate analysis of variance (ANCOVA) for disease manifestation at baseline-visit controlling for education as a co-variate in IBM SPSS Statistics V.28. Adjustment for multiple testing was applied using Bonferroni corrections. Dependent variables were tested for normal distribution using the Kolmogorov–Smirnov test (data not shown). Homogeneity of variances was asserted using Levene’s Test. Detecting unequal variances, values were reported with Welch’s test. Chi-square tests were used for analyses of categorical variables.

We additionally performed a 1:3 propensity-score matching controlling for the variables age, gender, education, CAG-high, disease duration, onset of HD motor symptoms, PBA-depression score and matched manifest control patients to those with sildenafil intake prior to baseline visit, having two more follow-up visits. Repeated measures ANOVA-analyses were conducted to determine longitudinal differences between those categories over 2 years. The longitudinal analysis of variance was assessed between groups at baseline and two more follow up visits as well as depicted as inner-subject parameters to observe the longitudinal progression.

## Data Availability

The data that support the findings of this study are available from the corresponding author upon reasonable request.

## References

[1] Fang J, Zhang P, Zhou Y (2021). Endophenotype-based in silico network medicine discovery combined with insurance record data mining identifies sildenafil as a candidate drug for Alzheimer’s disease. Nat Aging.

[2] Walker FO (2007). Huntington's disease. Lancet.

[3] Roos RAC (2010). Huntington's disease: a clinical review. Orphanet J Rare Dis.

[4] Komatsu H (2021). Innovative Therapeutic approaches for Huntington's disease: from nucleic acids to GPCR-targeting small molecules. Front Cell Neurosci.

[5] Wild EJ, Tabrizi SJ (2019). One decade ago, one decade ahead in Huntington’s disease. Mov Disord.

[6] Nabavi SM, Talarek S, Listos J (2019). Phosphodiesterase inhibitors say NO to Alzheimer's disease. Food Chem Toxicol.

[7] Liu L, Xu H, Ding S (2019). Phosphodiesterase 5 inhibitors as novel agents for the treatment of Alzheimer's disease. Brain Res Bull.

[8] Sanders O, Rajagopal L (2020). Phosphodiesterase inhibitors for Alzheimer's disease: a systematic review of clinical trials and epidemiology with a mechanistic rationale. J Alzheimers Dis Rep.

[9] Ibrahim MA, Haleem M, AbdelWahab SA (2021). Sildenafil ameliorates Alzheimer disease via the modulation of vascular endothelial growth factor and vascular cell adhesion molecule-1 in rats. Hum Exp Toxicol.

[10] Saavedra A, Giralt A, Arumí H (2013). Regulation of hippocampal cGMP levels as a candidate to treat cognitive deficits in Huntington's disease. PLoS ONE.

[11] Fusco FR, Paldino E (2017). Role of Phosphodiesterases in Huntington's Disease. Adv Neurobiol.

[12] Thakur T, Sharma S, Kumar K (2013). Neuroprotective role of PDE4 and PDE5 inhibitors in 3-nitropropionic acid induced behavioral and biochemical toxicities in rats. Eur J Pharmacol.

[13] Puerta E, Hervias I, Barros-Miñones L (2010). Sildenafil protects against 3-nitropropionic acid neurotoxicity through the modulation of calpain, CREB, and BDNF. Neurobiol Dis.

[14] Winder JY, Achterberg WP, Gardiner SL (2019). Longitudinal assessment of the Unified Huntington's Disease Rating Scale (UHDRS) and UHDRS-For Advanced Patients (UHDRS-FAP) in patients with late stage Huntington's disease. Eur J Neurol.

[15] Ribaudo G, Ongaro A, Zagotto G (2020). Therapeutic potential of phosphodiesterase inhibitors against neurodegeneration: the perspective of the medicinal chemist. ACS Chem Neurosci.

[16] Tipton PW (2020). Sexual dysfunction in Huntington's disease: what do we really know?. Neurol Neurochir Pol.

[17] Picillo M, Palladino R, Erro R (2019). The PRIAMO study: active sexual life is associated with better motor and non-motor outcomes in men with early Parkinson's disease. Eur J Neurol.

[18] Achenbach J, Saft C, Faissner S (2021). Longitudinal evaluation of the effect of tricyclic antidepressants and neuroleptics on the course of Huntington’s disease—data from a real world cohort. Brain Sci.

[19] Achenbach J, Saft C (2021). Data from ENROLL-HD: is the prevalence of juvenile and pediatric Huntington's disease overestimated?. Parkinsonism Relat Disord.

